# Relating behaviours and therapeutic actions during AVATAR therapy dialogue: An observational study

**DOI:** 10.1111/bjc.12296

**Published:** 2021-05-05

**Authors:** Conan O’Brien, Mar Rus‐Calafell, Tom KJ Craig, Philippa Garety, Thomas Ward, Rachel Lister, Miriam Fornells‐Ambrojo

**Affiliations:** ^1^ Research Department of Clinical, Educational and Health Psychology University College London UK; ^2^ East London NHS Foundation Trust London UK; ^3^ Mental Health Research and Treatment Center Faculty of Psychology Ruhr‐Universität Bochum Bochum Germany; ^4^ Department of Health Service and Population Research Institute of Psychiatry, Psychology & Neuroscience King’s College London UK; ^5^ Department of Psychology Institute of Psychiatry, Psychology & Neuroscience King’s College London UK; ^6^ South London & Maudsley NHS Foundation Trust UK; ^7^ North East London NHS Foundation Trust UK

## Abstract

**Objectives:**

AVATAR therapy is a novel relational approach to working with distressing voices by engaging individuals in direct dialogue with a digital representation of their persecutory voice (the avatar). Critical to this approach is the avatar transition from abusive to conciliatory during the course of therapy. To date, no observational study has examined the moment‐to‐moment dialogical exchanges of this innovative therapy. We aim to (1) map relating behaviours between participants and their created avatars and (2) examine therapeutic actions delivered within AVATAR dialogue.

**Method:**

Twenty‐five of the fifty‐three AVATAR therapy completers were randomly selected from a randomized controlled trial (Craig et al. *The Lancet Psychiatry*, **5**, 2018 and 31). Seventy‐five audio recordings of active dialogue from sessions 1 and 4 and the last session were transcribed and analysed using a newly developed coding frame. Inter‐rater reliability was good to excellent.

**Results:**

Fine‐grained analysis of 4,642 observations revealed nuanced communication around relational power and therapeutic activity. Early assertiveness work, reinforced by the therapist, focussed on increasing power and distancing. Participants’ submissive behaviours reduced during therapy, but the shift was gradual. Once the transition to a more conciliatory tone took place, the dialogue primarily involved direct communication between participant and avatar, focussing on sense of self and developmental and relational understanding of voices.

**Conclusions:**

AVATAR therapy supports voice‐hearers in becoming more assertive towards a digital representation of their abusive voice. Direct dialogue with carefully characterized avatars aims to build the voice‐hearers’ positive sense of self, supporting the person to make sense of their experiences.

**Practitioner points:**

- AVATAR therapy enables voice‐hearers to engage in face‐to‐face dialogue with a digital representation (‘avatar’) of their persecutory voice.
- Fine‐grained analyses showed how relating behaviours and therapeutic actions evolve during active AVATAR therapy dialogue.
- Carefully characterized avatars and direct therapist input help voice‐hearers become more assertive over the avatar, enhance positive sense of self, and support individuals to make sense of their experiences.

## Background

Auditory verbal hallucinations or ‘voices’ are highly prevalent among people with a diagnosis of schizophrenia, with around 70–80% experiencing them at first presentation (Goghari, Harrow, Grossman, & Rosen, 2013). They are associated with distress, reduced quality of life, and increased risk of suicide (Shergill, Murray, & McGuire, 1998; Hor & Taylor, 2010); with one in four failing to respond adequately to antipsychotic medication (Aleman & Laroi, 2011). Psychological interventions targeting distressing voices have evolved since the early nineties (Thomas et al., 2014). Voices are widely understood to form a continuum, ranging from subclinical hallucinatory experiences in the general population to those with associated distress and interference with life often occurring in the context of social adversity (Peters et al., 2016). Crucially, voices are viewed as social communicative acts (Bell, Mills, Modinos, & Wilkinson, 2017) rooted in relational patterns, beliefs about the self and others (Garety, Bebbington, Fowler, Freeman, & Kuipers, 2007; Morrison, 2001), and interpersonal trauma (Read, van Os, Morrison, & Ross, 2005). Submissive behaviour, a common response to perceived voice threats, maintains distress and a sense of entrapment (Birchwood, Meaden, Trower, Gilbert, & Plaistow, 2000), but, similar to other abusive relationships, voice‐hearers commonly report ambivalence about these relational experiences, including a sense of closeness and companionship (Valavanis, Thompson, & Murray, 2019).

How an individual responds to voices depends on their appraisals, including beliefs about power, control, and malevolence of the voice (Birchwood & Chadwick 1997; Chadwick & Birchwood, 1994). Cognitive behaviour therapy for psychosis targets these beliefs and has been found to reduce the overall severity of voices (see meta‐analyses in van der Gaag, Valmaggia, & Smit, 2014). A targeted cognitive therapy for command hallucinations (CTCH; Birchwood et al., 2014) has been found to reduce harmful compliance (Birchwood et al., 2018) by modifying power beliefs.

Relational approaches to voice hearing (Corstens, Longden, & May, 2012; Hayward, Overton, Dorey, & Denney, 2009; Leff, Williams, Huckvale, Arbuthnot, & Leff, 2013; Steel et al., 2019) have embraced the complexity and nuanced characterization of voices, foregrounding experiential techniques. In ‘Talking with Voices’ therapy (Corstens et al., 2012), the therapist talks directly to the voice, asking questions about the motives and origins of the voice, potentially reflecting conflict in the person’s life. Drawing from Relating Theory (Birtchnell, 1996, 2002), Relating Therapy (Hayward et al., 2009) explores parallels between social relationships and relating to voices, focussing on assertiveness training to address power beliefs. This 16‐session intervention pays attention to proximity (i.e., distance versus closeness to the voice) and its intersectionality with power. Role plays and ‘empty chair’ work (Chadwick, 2006) address unhelpful behavioural responses. A pilot randomized control trial showed large effect sizes in reduction of voice distress when compared to treatment as usual (Hayward, Jones, Bogen‐Johnston, Thomas, & Strauss, 2017). Lastly, in Making Sense of Voices, an approach developed in collaboration with voice‐hearers, voice dialoguing is focussed on understanding the meaning of voices in relation to life events with sessions over a 9‐month period (Steel et al., 2019).

The fourth main relational therapy for voices, and focus of the current paper, also uses a dialogical approach. In AVATAR therapy (Leff et al., 2013), voice‐hearers engage in face‐to‐face dialogue with a computer simulation (‘avatar’) of an audio‐visual representation of their voice experience. The therapy has been described elsewhere (Craig, Ward, & Rus‐Calafell, 2016; Leff, Williams, Huckvale, Arbuthnot, & Leff, 2014; Ward et al., 2020), but briefly, two treatment phases occur over six therapy sessions. Phase one focusses on encouraging individuals to be more assertive to the dominant voice while phase two begins with the avatar conceding power, becoming more dialogic and adopting a conciliatory position. The precise content of the dialogue with the avatar is informed by the individualized formulation and detailed characterization of the voice.

The efficacy of the therapy has now been demonstrated in two independent pilot studies (Leff et al., 2013; de Sert et al., 2018) and in a large fully powered randomized controlled trial comparing AVATAR therapy and supportive counselling that showed AVATAR therapy to be more effective post‐therapy in terms of reductions in the frequency, distress, and omnipotence of voices after an average of 6 therapy sessions (Craig et al., 2018). Results showed a large effect size (d = 0.8) in overall severity of auditory hallucinations in everyday life, which is substantially larger than in other trials targeting distressing voices (van der Gaag et al., 2014), although the difference between both arms was not sustained by 24‐week follow‐up, due to individuals in supportive counselling continuing to improve during that period.

In a recent report, Ward et al., (2020) offered the first comprehensive account of therapeutic targets in AVATAR therapy. Lead therapists in the trial conducted a systematic case review using session‐by‐session notes. Some therapeutic targets were clearly present for all therapy completers (*power and control*, *self‐esteem,* and *future focus*), whereas others (e.g., *maintenance processes*, *working with trauma*, *experiential disengagement*) were identified in some but not all participants, in line with the tailoring of the intervention to an individualized formulation. Despite the increased interest in mechanisms of action of AVATAR therapy and the potential value of examining the in‐session dialogue (Alderson‐Day & Jones, 2018; Hayward, 2018), no study to date has explored the evolving dialogue between voice‐hearer and avatar, nor looked at the specific therapeutic actions employed during these verbal exchanges.

The current study aims primarily to provide a detailed description of *observed* relating behaviours between the participant and constructed avatar and therapeutic actions during AVATAR therapy dialogue using a newly developed coding frame. We examine communicative acts as they navigate a shift from ‘controlling‐submissive’ to a ‘supportive’ relationship between voice‐hearer and their avatar. Understanding the detail of how the dialogue evolves in practice offers data triangulation (Patton, 1999) with adherence in this innovative intervention, but it is also crucial for future optimization and implementation of AVATAR therapy. This is because the AVATAR therapist not only develops a formulation to inform the direction of the therapy, but needs to think ‘on the spot’ and respond live as the characterized avatar.

Secondly, we aim to assess changes in relating behaviours and therapist contributions over time. As described elsewhere, the therapist aims to support and encourages the participant to face up to the avatar who speaks the verbatim content of the voice hearing experience at the initial stages. After the transition to a more conciliatory tone around session 4, the focus is then on the evolving dialogue between avatar and participant, requiring less active input from the therapist themselves. We wished to examine this empirically. The present study focusses on observable communicative acts during the active dialogue with the participant over time to evaluate whether the relational shift is delivered as intended. In particular, we hypothesized that (1) participant submissive and avatar controlling behaviours will decrease over time (i.e., session 1> session 4> last session) and (2) therapist contributions (number of vocal exchanges) during active AVATAR dialogue will decrease over the course of therapy.

## Method

### Background: AVATAR therapy provision within an RCT

This study formed part of the London‐based AVATAR RCT (Craig et al., 2018 ISRCTN65314790), and ethics approval were granted by the London‐Hampstead Research Ethics Committee, reference 13/Lo/0482. One hundred and fifty participants were randomly allocated to receive AVATAR therapy (*n* = 75) (the focus of the current paper) or supportive counselling (*n* = 75) (Craig et al., 2018).

### Participants

Twenty‐five individuals were randomly selected from the sample of 53 AVATAR therapy completers. This was to ensure that each participant had three audio recordings covering the beginning, middle, and end of therapy in order to capture therapeutic activity across therapy stages. Inclusion criteria to participate in the AVATAR RCT were as follows: (1) aged over 18 years; (2) troubling auditory hallucinations for at least 12 months; and (3) primary diagnosis of non‐organic psychosis (including ICD‐10 categories F20‐29 and F30‐39, subcategories with psychotic symptoms). Criteria for exclusion were as follows: (1) CBT for psychosis or attending a group specific to hearing voices; (2) unable to identify a single dominant voice to work on; (3) refusing all medication; (4) a diagnosis of organic brain disease; (5) a primary substance dependency; (6) auditory hallucinations not in English; (7) not having sufficient English language abilities to engage in therapy and assessments; and (8) not been able to complete primary outcome assessment measures.

### AVATAR therapy

**Figure 1:**
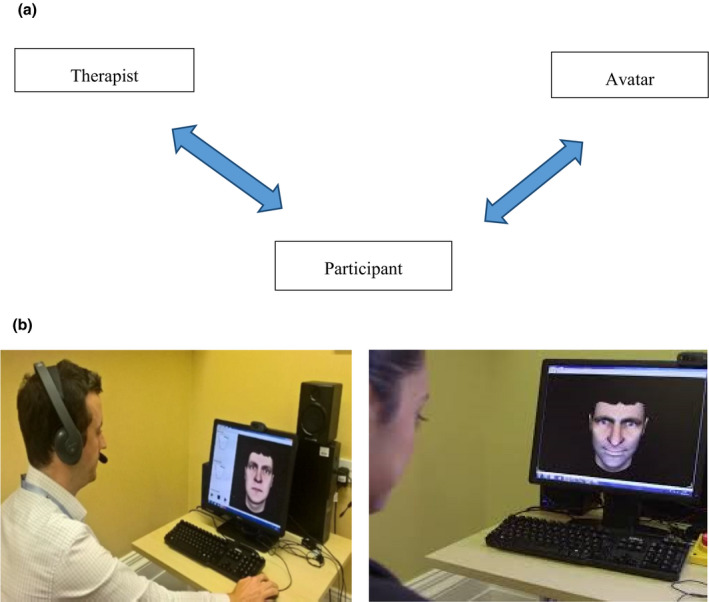
Active AVATAR therapy. (1a) Diagram displays the two dialogues during active AVATAR therapy, between the participant and the avatar and the participant and the therapist (i.e., there is no direct dialogue between the voice and the therapist). (1b) The left picture shows the therapist who switches between their own and the avatar voice to communicate with the participant, and the right picture shows the participant in front of the created avatar

The therapy was delivered over six weekly 50‐min sessions, of which 5–15 min involves face‐to‐face work with the avatar, wherein the therapist facilitates a direct dialogue between the participant and the constructed avatar (the ‘avatar’ is voiced by the therapist using voice transformation software). Therapy evolves through two phases: Phase one (typically sessions one to three) focusses on exposure to the avatar speaking distressing verbatim content of the participant voices, while the therapist encourages assertive responding (see Figure 1); and in phase two (typically sessions four to six), the dialogue gradually evolves as the avatar concedes ground and acknowledges the strengths of the participant. Therefore, therapeutic actions are initially employed by the therapist to support the participant to manage anxiety and stand up to the avatar, but once transition to phase two occurs, the avatar himself/herself has a therapeutic role within its enacted characterized voice, targeting processes that are specific to an individualized formulation, such as self‐esteem enhancement and making sense of voice experiences.

Training involved 1‐2 closely supervised pilot cases with Prof Julian Leff using the AVATAR therapy manual. The 5 trial therapists were skilled clinicians with at least 5 years of clinical experience, and who were competent in using the cognitive‐behavioural therapy approach (Craig et al., 2018; Ward et al., 2020). Their professional background was clinical psychology (3), psychiatry (1), and counselling psychologist (1).

### Procedure and measures

Baseline measures from the AVATAR RCT used to characterize the sample included the *Psychotic Symptom Rating* *Scale –* *Auditory Hallucinations* (PSYRATS‐AH; Haddock, McCarron, Tarrier, & Faragher, 1999); the *Voice Power Differential* *Scale* (VPDS; Birchwood et al., 2000); and the omnipotence and malevolence sub‐scales of the *Beliefs About Voices Questionnaire‐Revised* (BAVQ‐R*;* Chadwick, Lees, & Birchwood, 2000).

All AVATAR therapy sessions were audio‐recoded with participant consent. Therapy sessions 1, 4, and last were selected for the current study to ensure that the avatar transition in character and way of relating was captured. The avatar dialogic shift and reduction in hostility is timed by the therapist (based on individual’s formulation) but typically occurs around session 4. At this stage, the avatar became more conciliatory, supportive, and respectful (Craig et al., 2016; Leff et al., 2014).

The focus of the current study was on the active dialogue between participant/avatar/therapist – the pre‐ and post‐dialogue debriefing was not included. Seventy‐five AVATAR therapy sessions of 25 participants were transcribed by the first author. For the purpose of developing the coding frame (see below), a coding unit was defined as a vocal exchange between participant/avatar/therapist (e.g., ‘Leave me alone and don’t come back’).

### Development of coding frame

A coding frame was developed in line with the study’s objectives and hypotheses in order to capture both a) relating behaviours between participant and avatar where power and control are negotiated and b) therapeutic actions used during AVATAR therapy.

As is typical in the development of coding systems (Bakeman & Gottman, 1997; Pope, Ziebland, & Mays, 2000), an iterative process was followed. In *stage I* *(Identifying a thematic framework)*, the literature on cognitive and interpersonal theories and therapies for voice hearing [Phenomenology of voice hearing (McCarthy‐Jones et al., 2014); Beliefs about voices (Birchwood et al., 2004; Chadwick & Birchwood, 1994); CBT for psychosis (Garety, Kuipers, Fowler, Freeman, & Bebbington, 2001; Morrison, 2001); Relating Therapy (Birtchnell, 1996; Hayward et al.,^2009^, ^2017^); Talking with voices therapy (Corstens et al., 2012); and other voice focussed therapies (Thomas et al., 2014)] was reviewed and a list of potential voice relating behaviours and therapeutic actions extracted. In *stage II* *(familiarization)*, AVATAR literature was reviewed (Craig et al., 2016; Leff et al., 2014), AVATAR therapy pilot recordings were listened to, and the AVATAR therapy clinical trial manual was examined (Craig et al., 2018) to inform coding development. Preliminary codes were discussed in *stage III* in a consensus meeting with AVATAR trial therapists and principal investigators of the AVATAR RCT (Craig et al., 2018). During *stage IV,* we followed Green et al., (2006) method where a sample of transcripts were selected and analysed in order to ensure that key themes, relating behaviours, and therapeutic actions were captured. Categories were refined and new codes added if necessary. In the *fifth stage*, five hundred and forty‐five coding units belonging to six randomly selected transcripts (two from session 1, two from session 4, and two from the last session) were coded by two of the authors to assess inter‐rater agreement for main codes (four *relating behaviours* and five *therapeutic actions*). As seen in Table 1, Kappa values were indicative of near perfect agreement (Landis & Koch, 1977).

**Table 1:** Inter‐rater agreement

Codes	Kappa
Relating behaviours	
Controlling (avatar)	.83
Autonomy enabling (avatar)	.86
Submissiveness (participant)	.82
Assertiveness (participant)	.89
Therapeutic techniques	
Emotional Attunement	.87
Enhancing Power & Control	.92
Relational & Developmental Understanding of Voices	.88
Self‐esteem	.83
Hope & Future Orientated	.92

### Quantitative analyses

All data were analysed using the statistical package IBM statistics 21 SPSS. To assess change in relating behaviours and in vocal exchanges, independent repeated measures ANOVA was conducted for parametric data and Friedman test for non‐parametric data. An alpha level of.05 was used for statistical significance. Post‐hoc analyses were carried out for all significant findings. Paired Wilcoxon signed rank tests were conducted to assess differences between sessions.

## Results

### Demographic, clinical characteristics, and session length

Table 2 shows that the twenty‐five participants included in the current study were predominantly male with a diagnosis of paranoid schizophrenia, with over half of the sample belonging to a non‐White British ethnic group.

**Table 2:** Demographic and baseline clinical characteristics

	AVATAR therapy arm (*n* = 75)		Test
	In the current study (*n* = 25)	Not in the current study (*n* = 50)	
Demographic			
Age (years): Mean (*SD*)	43.36 (9.20)	42.02 (10.64)	*t*(73) = −.54, *p* = .57 95% CI: −6.31–3.63
Gender (male): *n* (%)	18 (72%)	39 (78%)	*x* ^2^ (1) = .33, *p* = .57
Ethnicity: *n* (%)			
White British	9 (36%)	17 (34%)	*x* ^2^ (2) = .11, *p* = .95
Black Ethnicity^1^	9 (36%)	20 (40%)	
Other Ethnicity^2^	7 (28%)	13 (26%)	
Clinical (general)			
Paranoid schizophrenia *n* (%)	20 (80%)	37 (74%)	*x* ^2^ (1) = .33, *p* = .57
Duration of illness (years): Mean (*SD*)	24.56 (10.42)	18.44 (10.76)	*t*(73) = −2.34, *p* = .02* 95% CI: −11.32 to −.92
Voice specific			
Number of voices: Mean (*SD*)	4 (3.21)^a^	2.46 (1.64)^b^	*t*(58) = −2.47, *p* = .02* 95% CI: −2.79 to −.29
PSYRATS –AH Total: Mean (*SD*)	28.44 (4.42)	30.04 (4.81)	*t* (73) = 1.36, *p* = .84 95% CI: −.68 to 3.68
VPDS Total: Mean (*SD*)	21.52 (6.55)^c^	21.86 (6.88)^d^	*t* (55) = .18, *p* = .78 95% CI: −3.38 to 4.05
BAVQ‐R Mean (*SD*)			
Malevolence Total	10.32 (3.92)	10.84 (4.91)	*t* (73) = .46, *p* = .84 95% CI: −1.73 to 2.77
Omnipotence total	9.88 (4.27)	10.34 (3.84)	*t* (73) = .47, *p* = .25 95% CI: −1.48 to 2.41

^1^ Black British, Black Caribbean, and Black African.

^2^ Asian Indian, Asian Chinese, and other. Excluding participants who reported an uncountable number of voices.

^a^ *n* = 21.

^b^ *n* = 39 (these percentages were 16% and 22%). The equivalent value of unaccountable number of voices in McCarthy‐Jones et al., (2014) phenomenological study was 28%. Completed VPDS.

^c^ *n* = 21.

^d^ *n* = 36.

* *p* < .05.

Voice hearing experiences reflected what has been reported elsewhere (Birchwood’s et al. 2018; Hayward et al., 2017; McCarthy‐Jones et al., (2014). Hearing more than one voice was common, hallucinatory experiences were in the severe range, and participants reported diminished power in relation to voices. Participants in the current study had a longer duration of illness than those randomized to receive AVATAR therapy but not included in the study. There were no other significant differences between the groups.

### Relating behaviours

We observed 2,442 relating behaviours between participants and their avatars (1,261 participant vocal exchanges) over the active dialogue section of three therapy sessions (sessions 1, 4, and last). The coding frame for relating behaviours mapped the power, control, and proximity between participant and avatar during this active dialogue. Table 3 provides descriptions, verbatim examples, and number of coded behaviours for each behaviour in the four main relating codes: controlling avatar, submissive participant, encouraging of autonomy avatar, and assertive participant.

**Table 3:** Relating behaviour between the avatar and the participant with verbatim examples (total number observed utterances per category)

	Avatar	Participant
Control‐submission	Controlling **Abuse/insult/negative evaluation** of other: Mocking, ridiculing, name‐calling. ‘*You’re ugly and useless’. (175)* **Holding on/reluctance to change relationship style:** Resistance to change relationship dynamic. ‘*But I need to be in your life’. (93)* **Undermine (instil doubt**): To instil doubt in other and maintain dominance. ‘*You’re putting on a show because that’s what that doctor told you to do’. (91)* **Demand:** Instructs other to act. ‘*You must take the drugs’. (34)* **Threat (psychological):** Threat of psychological harm. ‘*I don’t mind if I do cause you grief’. (34)* **Threat (physical):** Threat of physical harm. ‘*I’m going to kill you tomorrow morning’.(26)*	Submissiveness **Helpless/ over‐reliance on others:** Includes reliance on other/belief that can’t help self. **‘** *…it’s not very nice for me to have to throw them away but since you’re saying I have no other choice*’.(195) **Speechless/hesitant:** Voice‐hearer comes across as uncertain. ‘*Oh…em*…’ (80) **Appeasement:** Conciliatory response. ‘*I might be an idiot but I’m a careful one*’. (36) **Fears about ending the relationship:** Reticence about ending relationship with avatar. ‘*I’m pleased in one way and in another way, I’m going to miss you*’. (20) **Request advice/guidance:** Places other in expert position. ‘*Well, what do I do*?’ (17)
Autonomy Enabling‐ assertiveness	Autonomy enabling **Negotiate/move towards emancipation**: A shift in relating style. ‘*Well if you continue like this I will be fading from your life*’. (250) **Concession of power:** Acknowledgement no longer as powerful/ able to control. ‘*I see. Well if I’m honest I think you’ve already started to take control back from me*’. (164) **Acknowledgement** **of change in the relationship with voices/ avatar**: How the participant manages avatar/voices. ‘I think you are changing, you’re accusing me’. (125) **Curiosity:** About relational change, showing a desire to elicit more detail about new position. ‘*What do you mean?’* (117) **Advice giving:** Expert position/ mentors. ‘…*I think it just puts a lot of pressure on you and then, you know what, maybe you feel a little bit less confident’*. (72)	Assertiveness **Challenge/dismiss other’s assertion**: Disagrees with other. ‘*You’re not better than me’. (224)* **Separate – distance**: Preference for distance, personal space/ privacy. ‘*I want you to go away, stay out of my life because you don’t own me’. (222)* **Self‐agency:** Re‐establishes interpersonal control. ‘*I’ll say what I like’. (166)* **Ending of relationship**: Informs ending of ‘relationship’. ‘*Think I’m ready to follow that plan [of no longer speaking with voices] to see how it goes’. (93)* **Increase power:** A shift from powerless to powerful. ‘*Yes I believe I’ve taken the power away from you’. (81)* **Downplays threat/impact**: Minimizes threat. ‘*You’re not having the effect that you use to have on me. I’m able to ignore you more now and carry on*…*’ (67)* **Separate – disaffiliate:** States that one is separate/ different from other. ‘*You’re… you’re not like me, you’re more negative than me’. (60)*

During the *control‐submission mode* of relating, the avatar displayed a range of *controlling behaviours*, the most common being emotional abuse, such as name‐calling, followed by attempts to instil doubt and retain dominance. Specific commands and threats were relatively less frequent, possibly because the AVATAR therapy protocol did not permit the use of direct commands to hurt oneself or others (Ward et al., 2020). Participant *submissive* behaviours reflected lack of power, passiveness, and uncertainty. The most common type of utterance was of helplessness, reflecting a belief that the participant had no choice but to follow the avatar’s lead.

On the contrary, *assertive participant behaviour* involved predominantly active challenging or dismissal of the avatar’s critical remarks, indicating increased self‐agency and control. Around one quarter of assertive utterances also related to a wish to separate from/end the relationship with the avatar. *Enabling autonomy* avatar behaviours included a transfer of power and control (targeting beliefs about voices), and inviting participants into a new relational space.

### AVATAR therapy: Relating behaviour hypotheses

Table 4 shows the changes in relating behaviours over the course of therapy. As hypothesized, avatar controlling behaviours significantly decreased between sessions 1 and 4, remaining low until the end of therapy. Participant submissiveness however showed a more gradual change. The overall significant decrease between the first and last sessions was attributable to reductions during the second half of therapy.

**Table 4:** Observed participant and avatar relating behaviours during AVATAR dialogue

	Median [Range]			Main test	Post‐hoc tests		
	S1	S4	Last		S1 – S4	S4 – Last	S1 – Last
Avatar – Controlling	15 [7–28]	0 [0–37]	0 [0–5]	*x* ^2^(2) = 37.18, p = <.001**	*z* = −3.97, *p* < .001**	*z* = −1.65, *p* = .09	*z* = 4.38, *p* < .001**
Participant –Submissiveness	4 [0–29]	3 [0–29]	1 [0–23]	*x* ^2^(2) = 7.52, *p* = .02*	*z* = −.39, *p* = .70	*z* = −2.33, *p* = .02*	*z* = −2.76, *p* = .01*
Avatar – Autonomy Enabling	7 [1–13]	15 [1–34]	1 [2–19]	*x* ^2^(2) = 9.94, *p* = .01*	*z* = −3.31, *p* < .001**	*z* = −2.63, *p* = .01*	*z* = −1.60, *p* = .11
Participant –Assertiveness	17 [5–23]	10 [0–70]	9 [0–26]	*x* ^2^(2) = 16.64, *p* = < .001**	*z = −*3.13, *p* = .01*	*z* = −.58, *p* = .56	*z =* −3.69, *p* < .001**

* *p* < .05,

** *p* < .001.

Exploratory analyses on the assertive‐autonomy mode revealed that participant assertive behaviours significantly diminished between sessions 1 and 4, showing an overall reduction between session 1 and last session. The avatar actions designed to enable participant autonomy significantly increased between sessions 1 and 4, followed by a significant reduction between sessions 4 and last, with an overall lack of change between the first and last sessions. This pattern illustrates the planned evolution of AVATAR dialogue, reflecting a shift from a battle for power and dominance between participant and avatar in the initial phase of therapy to a shift around session 4 to a relationship that appeared more collaborative, and therefore does not require ongoing assertions from the participant to make their needs heard.

### Therapeutic actions during AVATAR dialogue

The coding frame for the range of therapeutic activity identified is presented in Table 5. Eighteen therapeutic techniques were grouped in five categories: emotional attunement, enhancing power and control, relational and developmental understanding of voices, self‐esteem, and lastly, hope and future oriented actions. We coded 2,200 therapeutic actions. As mentioned in the method, once the transition from verbatim persecutory content to conciliatory tone occurred (usually by or around session 4) most interventions were delivered through direct dialogue with the avatar. For example, the avatar helped the voice‐hearer to make connections with past experiences or emphasized positive changes and instilling hope. This often involved the avatar’s own reflections about having been previously mistaken about the participant (e.g., ‘*I have misjudged you’*) or explaining his inner motives (e.g., ‘*I’m just an echo of the bad things you’ve heard said to you*’).

**Table 5:** Therapeutic actions

Code (Total)	Description *Verbatim examples (Num. observed utterances; Num. participants)*
Emotional attunement (212)	**Check in (emotional state, distress, coping)**: Therapist checks in with participant. ‘*Just want to check in again how you feeling?’* *^T^ (142, 24)* **Empathy**: An empathic response to participant’s feelings/behavioural responses to voice/events. ‘*Well I know you’ve been battling a long time but let’s just keep strong…they think they’re ahead of you but they’re not’*. *^T^ (57, 12)* **Normalizing:** Therapist normalizes participant responses to voices/events/therapy. ‘*That’s ok. You’re supposed to be nervous, he’s a nasty bully’*. *^T^ (13, 7)*
Enhancing power and control* (677)	**Reinforce:** Participant opposes avatar and therapist reinforces assertive behaviour. ‘*That’s really good. You’ve done really well; you’ve got lots of positive stuff here*’. ^T^ (236, 24) **Encouragement**: Therapist continues to encourage participant/ advices on how to be assertive/deal with avatar. ‘*And I want you to, make it, and mustn’t let him interrupt you…take command of the situation, alright?*’ ^T^ (147, 24) **Verbatim instruction:** Therapist delivers a direct instruction. *‘Say ‘Now I’m going to leave you alone, I’m not going to listen to you anymore. 10 years is enough’. Ok?’* ^T^ (137, 24) **Participant invited to decide direction of the dialogue**: Allows for participant autonomy. ‘*What do you want to say to me today?*’ ^A^ (157, 24)
Relational & developmental understanding of voices(525)	**Reflection on own/other behaviour/** inner world. Avatar explains own behaviour/ way of thinking. *‘I thought you need me, I thought you needed to hear the things that I said*’. ^A *‘*^ *I get a bit scared as I try to come across more confident than I am to … cover up’*. ^A^ (288, 24) **Changeability (of one’s and/or other’s internal world, thoughts, feelings):** Representation of self and others internal world as changeable; that one’s opinions can change. ‘*And I wonder, the picture I’m getting of you is very different. As I said I have misjudged you’*. ^A^ (114, 21) **Voices linked to inner beliefs:** Content link with inner beliefs about the self ‘I’ve been echoing some of the things that you think about yourself. You’ve called yourself useless and worthless’. ^A^ (54, 18) **Voices as internally generated beliefs:** Voices as internally generated (attribution of source). *‘I’m only here when you feel bad*’. ^A^ (41, 14) **Biographical context (including trauma and loss**): Explicit linking voice experiences to past experiences and/or relational development. *‘I’m just an echo of the bad things you’ve heard said to you’*. ^A^ (28, 14)
Self‐Esteem (528)	**Enquiry about positive qualities/other’s views/ functioning**: Line of questioning to improve positive self. ‘*What do you make of the list X has written for you?’* *^A^ (289, 24)* **Positive evaluation of other:** agreeing with other’s attributes/self‐praise/strengths. ‘*Yes you’ve made a heroic achievement in your life’*. *^A^(239, 24)*
Hope & future oriented (258)	**Goal setting/ behavioural specific goals:** Identification of activities and goals to work towards. ‘*That’s all quite a little way ahead but what you do in the next few weeks?’* *^A^ (66, 17)* **Positive statement on recovery:** Communicating progress/ success. ‘*… it’s good to hear that you’ve not been getting any bullying [voices] in the last week’*. *^A^(30, 11)* **Problem Solving about voices:** Solutions for future hypothetical situations. ‘*So what will you do if you hear me again, will you stumble?’* *^A^ (54, 14)* **Instil hope**: Well‐wishing regarding continued broader successes and recovery. ‘*That’s all quite a little way ahead but what will you do in the next few weeks?’* *^A^ (108, 22)*

A = avatar and T = therapist. * Therapist only code as it involves encouraging the participant to stand up to the avatar.

**Figure 2:**
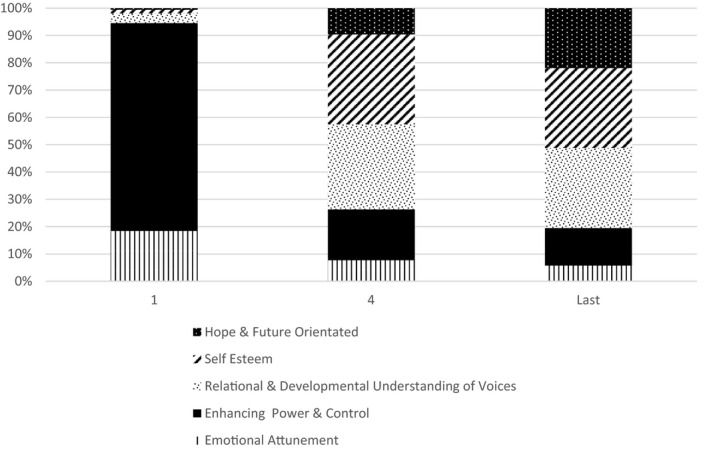
Percentage breakdown of each therapeutic technique observed per therapy session

Overall, the most frequently used communicative therapeutic actions related to ‘enhancing power and control’ work, followed by the ‘self‐esteem and ‘relational and developmental understanding of voices’ categories. A lower number of verbal exchanges related to hope/future oriented statements and lastly emotional attunement/engagement. The picture however did vary across therapy sessions. Figure 2 illustrates the percentage breakdown of each therapeutic actions delivered in sessions 1, 4, and last of active AVATAR therapy dialogue. During session 1, 76% of observed therapeutic actions focussed on promoting participant power and control over the avatar. Examples include ‘*that’s brilliant, keep going like that’* and included verbatim suggestions of what to say to the avatar ‘*tell him you’re not getting bullied by him and that it’s your life’*. Emotional attunement such as therapist checking in with the participant made up around 18% of observed therapeutic techniques during session 1.

There is a shift in focus from session 4 involving a substantial reduction in the use of therapist’s encouragement designed to increase participant’s power and control over the avatar. Instead, there is an increase in techniques designed to improve participant’s sense of self and understanding of voices. Specifically, in sessions 4 and last, 33% and 29%, respectively, of therapeutic actions related to enhancing self‐esteem, for example, avatar saying ‘*…you have creativity and talent. Such a gift*’. Similarly, relational and developmental understanding of voices made up 31% and 29% observations in sessions 4 and last, which typically included reflecting on the inner world. For example, the avatar letting the participant know the reasons why they were being abusive. Lastly, hope and future planning techniques were overall less frequently used, increasing to a maximum of 22% of verbal exchanges in the last reported session. Examples included goal setting and problem solving future hypothetical situations.

As can be seen in Table 5, examples of the various therapeutic actions were found in the active dialogue sessions for most participants (e.g., verbatim instructions to enhance power and control were reported in 137 observed utterances in 24 of the 25 participants included in the current study). Only 3 of the therapeutic actions (empathic statements, normalizing and positive statement about recovery from voices) were only present in less than half of the participants (*n* < 13).

### Therapist diminishing active support hypothesis

Table 6 displays information on the number of vocal exchanges of face‐to‐face AVATAR therapy dialogue. These sessions averaged approximately 10 min, and participants had the highest frequency of exchanges across all three sessions. As hypothesized, we found a significant reduction in direct therapist input after the first session (see Table 6). Participant contributions significantly increased after session 1 and remained at that level by the last session, whereas avatar’s number of exchanges increased in session 4 but then decreased.

**Table 6:** Observed number of vocal exchanges during AVATAR dialogues

	Median [Range]			Main Test	Post‐hoc Tests		
	S1	S4	Last		S1 – S4	S4 – Last	S1 – Last
Therapist	20 [8–34]	8 [0–21]	5 [0–31]	*x* ^2^(2) = 35.64, *p* = <.001**	*z* = −4.25, *p* < .001**	*z* = −2.43, *p* = .02*	*z* = −4.19, *p* < .001**
Avatar	25 [9–41]	51 [17–117]	41 [12–82]	*x* ^2^(2) = 31.17, *p* = < .001**	*z* = −4.32, *p* < .001**	*z* = −2.45, *p* = .01*	*z* = −3.83, *p* < .001**
Participant	41 [10–62]	59 [21–123]	49 [16–84]	*x* ^2^(2) = 10.97, *p* = <.001**	*z* = −3.71, *p* < .001**	*z* = −3.27, *p* < .001**	*z* = −1.77, *p* = .08

Please note that session length (in minutes) varied with the first session being the shortest (mean (*SD*) = 7.3 (2.4), range [3.3–13.1]), with session 4 the longest (mean (*SD*) = 13.3 (4.5), range [6.1–22.3]), and with the final session falling in between ((*SD*) = 10.1 (5.8), range [3.5–28.2]).

* *p* < .05.

** *p* < .001.

## Discussion

The current study focussed on the dialogue between voice‐hearer, a computer simulation of their voice experience (‘avatar’), and the therapist during the active part of AVATAR therapy. The development of a coding frame enabled a fine‐grained analysis of what actually happens during active AVATAR dialogue over the course of therapy. Inter‐rater agreement was good to excellent. As hypothesized and consistent with AVATAR therapeutic aims, results show that avatar controlling behaviours decreased significantly between sessions 1 and 4, remaining low until the end of therapy. Furthermore, direct input and therapeutic actions coming from the therapist reduced significantly and progressively after session 1, when the need to check in with the client when she/he first faces the avatar is at its highest. The observed relating behaviours between voice‐hearer and avatar and the therapeutic actions employed were delivered as intended over two phases, adding further support to reports of high fidelity and overall adherence to the manual (Craig et al., 2018). Please also see Table S1 for a mapping of observed communicative acts during avatar dialogue against specified avatar therapy targets (Ward et al., 2020).

### Claiming power in an abusive relationship

AVATAR therapy aims to increase hearer power and reduce voice dominance (Craig et al., 2018; Leff et al., 2014). Distressing voices are typically characterized by negative content and affective consequences (Beavan & Read, 2010; Close & Garety, 1998; Nayani & David, 1996). AVATAR therapy provides a realistic simulation of the persecutor’s voice heard by the person during everyday life, which seems to be crucial to important therapy outcomes (Rus‐Calafell et al., 2020). Although direct use of abusive verbatim voice content presents challenges, accurately representing voice content provides an opportunity for validation of experiences and may facilitate habituation (Rus‐Calafell et al., 2020; Ward et al., 2020).

On the other side of the relationship, participant’s submissive behaviours, which include helplessness, tentativeness when facing the avatar’s controlling behaviour, and appeasement, do not substantially reduce until the end of therapy. At the start of AVATAR therapy, the therapist active contribution is at its highest, with a focus on enhancing participant’s power and control, which ranges from reinforcing participant’s hesitant attempts to be assertive towards the avatar to offering direct instructions. Accordingly, participant assertive behaviours are at their highest during session 1, where attempts to challenge and dismiss avatar’s controlling behaviour (such as threats and negative evaluations) are as frequent as expressing a need to separate/ distance oneself from the relationship with the avatar. These findings mirror other relational therapies, highlighting the need to assert one’s needs and set interpersonal boundaries (Corstens et al., 2012; Hayward et al., 2014; Steel et al., 2019). General implications for psychological therapies for people who hear distressing voices, such as CBT for psychosis, include reflecting on the wide range of submissive behaviours displayed during dialogue with an (embodied) voice, and the high frequency of therapist encouragement and support needed for voice‐hearers to increase control over their voices and assert their own needs.

The above observations on the vocal exchanges during AVATAR active dialogue are in line with outcome data in the RCT (Craig et al., 2018), where, by the end of the intervention, endorsement of omnipotence about voices had significantly reduced and a trend was identified for an overall increase in subjective assertiveness towards the voice.

### Conciliatory avatar dialogue: enhancing sense of self and understanding the voice

The current study illustrates that AVATAR therapy clearly goes beyond assertiveness work. By session 4, once the avatar has transitioned from being abusive to conciliatory, the participant’s need to assert power and separate from the avatar is diminished, and accordingly, the need for the therapist to actively encourage the participant to take control over the avatar. This is also reflected in an overall reduction of therapist’s contributions during active avatar dialogue as therapy progresses.

During this second phase, the active dialogue is therefore between the participant and the avatar and it predominantly focusses on addressing the person’s sense of self and helping them to make sense of their voice. This is in line with existing cognitive models of voices (Chadwick & Birchwood, 1994; Garety et al., 2007; Morrison et al., 2001), but what is unique about AVATAR therapy and other relational approaches (Corstens et al., 2012; Hayward et al. 2014) is that the work is done experientially, echoing abusive relationships in the person’s life. Each avatar is characterized to portray the participants’ adverse voice experiences, and it is in the context of this evolving relationship that the avatar eventually shows appreciation for the positive qualities of the participant and nurtures self‐agency in their recovery. Similarly, the avatar talks to the participant about the reasons why they behaved abusively in the past and their own shortcomings. While these therapeutic dialogic strategies help rebalance the power in the relationship with the avatar (Craig et al., 2016), they also arguably resemble mentalization approaches, as proposed by Brent and Fonagy (2014), as the avatar reflects on the changeability on one’s internal world. Accordingly, these interactions might facilitate participants’ ability to infer their own and others mental states (Bateman & Fonagy, 2004).

Specific links to current and past autobiographical context, including trauma, were observed in over half of the sample during active AVATAR therapy dialogue, in line with Ward et al. (2020)’s description of the ‘working with trauma’ target in (36% ‘clearly’ and an additional 38% ‘partially’), the total sample of therapy completers (*n* = 53). Whereas Ward et al., (2020) offer an overview of how AVATAR therapy is conceptualized in its entirety, the current study focussed on verbatim communicative acts analyses at the microlevel within 3 sessions. It is therefore of note that in the absence of information about the intentions of the therapist, statements such as reflecting on voices and inner beliefs were coded within the ‘voices linked to inner beliefs’ category, but they might in fact have been specifically linked to traumatic events outside the active dialogue. Further, the content of distressing of voices is not always directly linked to previous traumatic experiences (Hardy et al., 2005), making it potentially less likely to be observed during active AVATAR therapy dialogue.

### Limitations

Participants included were only those who completed the course of the therapy, and therefore, it remains an open question whether the findings are representative of those who disengaged from therapy. Although the subsample was selected using simple random sampling, and in general representative of the whole cohort, they did present with a longer duration of illness. It is possible that the content of active dialogue of individuals with shorter histories, such as those with first episode psychosis, could differ. The study also focussed on a fine‐grained analysis of the active avatar dialogue and did not explore any therapeutic work conducted during preparatory and post‐dialogue discussions. Crucially, the current study revealed a planned evolution of AVATAR dialogue, where voice‐hearers’ utterances were not independent, but in fact a response to the therapist led changes in the avatar utterances. Therefore, the current paper does not offer a naturalistic observation of how dialogue might spontaneously evolve between voice‐hearers and everyday voices, but instead supports data triangulation with AVATAR therapeutic aims (Ward et al., 2020) and adherence (Craig et al., 2018). Moreover, only three out of the six AVATAR dialogue sessions were analysed in the current study, so it is possible that a detailed examination of sessions within phase I (sessions 1‐4) could have revealed more subtle changes on voice‐hearers’ submissive responses in the context of hostile avatar behaviour and varied voice‐hearer’s trajectories.

The observational analyses reported here do not capture other relevant aspects of experience of AVATAR therapy, such as the level of characterization of the targeted voice or the voice‐hearers’ subjective experience of navigating a dialogue with an initially abusive avatar that becomes supportive. The latter will be reported in a forthcoming qualitative paper as mentioned in the trial protocol (Craig et al., 2015).

### Future directions

The current observational study highlights the importance of evaluating how the voice‐hearer experiences facing up to the avatar as therapy evolves, given the observed very gradual reduction of submissive behaviours, and their range, from appeasement, expressing fears about ending the relationship, to being hesitant and speechless, which links to their subjective reports of anxiety during sessions (Rus‐Calafell et al., 2020). Voice‐hearers face a challenging task that requires therapist attunement, support, and encouragement to gain power and control over their voice. Further research should investigate the role of therapeutic alliance in AVATAR therapy, given its associations with outcome in psychological therapies for psychosis (Bourke, Barker, & Fornells‐Ambrojo, 2021).

An important question is whether both phases of AVATAR therapy are necessary for all individuals (Craig et al., 2018). Voice‐hearers might benefit from AVATAR therapy primarily because of the opportunity to habituate to, and assert their needs to a persecutory voice under the guidance of a well‐attuned therapeutic relationship, or it could be that specific therapeutic actions, such as unfolding relational and developmental understanding of voices during the dialogue, are fundamental to address distress. A new trial (http://www.isrctn.com/ISRCTN55682735) is planned to examine this question and explore moderators of treatment efficacy, such as complexity of characterization of the dominant voice, which we already know is associated with engagement in voice dialogue (Ward et al., 2021).

The current study provides the first empirical investigation of the planned evolution of the dialogues in AVATAR therapy. This fine‐grained analyses of relating behaviours and therapeutic actions within avatar dialogue provide insights which will inform ongoing attempts to optimize and personalize the AVATAR therapy approach.

## Conflict of interest

All authors declare no conflict of interest.

## Supporting information

Table S1. Correspondence between coded communicative acts during avatar dialogue.

## Data Availability

Anonymized data sets available on reasonable request to the senior author with a written proposal for the reason for the request.
