# Supplementary material for: Relating behaviours and therapeutic actions during AVATAR therapy dialogue: An observational study
Source: Br J Clin Psychol. 2021 May 5;60(4):443–62. doi: 10.1111/bjc.12296 (PMC12086748; doi:10.1111/bjc.12296)
Supplement: Supplementary file 1 — Table S1. Correspondence between coded communicative acts during avatar dialogue. [file BJC-60-443-s001.docx]

Table S1 Correspondence between coded communicative acts during avatar dialogue (relating behaviours and therapeutic actions) and AVATAR therapy targets as described in Ward et al. (2020)

| Type | Code | Description | Therapeutic target in AVATAR therapy (Ward et al., 2020) |
| --- | --- | --- | --- |
| Relating behaviours | Controlling | Abuse, reluctance to change relationship, undermine, demand, threat | Power and control* |
|  | Submissive | Over-reliance, hesitant, appeasement, fears about ending relationship, request advice | Power and control* |
|  | Autonomy enabling | Negotiate emancipation, concession of power, acknowledge change in relationship, curiosity, advice | Power and control* |
|  | Assertive | Challenge/dismiss, separate, self-agency, ending relationship, increase power, downplay threats, disaffiliate | Power and control*  Experiential Disengagement |
| Therapeutic actions | Enhancing power and control* | Reinforce, encourage, verbatim instruction, invited to decide direction of the dialogue | Power and control* |
|  | Engagement & attunement | Check in, empathy, normalise | *-* |
|  | Relational & developmental understanding of voices | Reflection on own/other behaviour/ inner world | Maintenance processes*  Identity/social inclusion*\|  Compassion to voice* |
|  |  | Changeability (of one’s and/or other’s internal world, thoughts, feelings) | Compassion to voice*  Identity/social inclusion*\| |
|  |  | Voices linked to inner beliefs | Working towards internal attribution*  Maintenance processes*  Working with trauma* |
|  |  | Voices as internally generated beliefs | Working towards internal attribution |
|  |  | Biographical context (including trauma and loss) | Identity/social inclusion*\|  Working with grief  Working with trauma* |
|  | Self Esteem | Enquiry about positive qualities/other’s views | Self Esteem/ Self concept |
|  |  | Positive evaluation of other |  |
|  | Hope and future oriented | Goal setting/ behavioural specific goals | Maintenance processes*  Future focus |
|  |  | Positive statement on recovery from voices |  |
|  |  | Problem Solving about voices |  |
|  |  | Instil hope |  |

*Therapeutic target potentially addressed by more than one type of communicative act (relating behaviour/ therapeutic action)
